# Hematological Indices in COVID-19 Patients with Rhinosinusitis Mucormycosis

**DOI:** 10.22038/IJORL.2024.75276.3525

**Published:** 2024-03

**Authors:** Seyedeh Sabereh Mojtahedi, Hossein Zarrinfar, Mehdi Bakhshaee

**Affiliations:** 1 *Department of Parasitology and Mycology, School of Medicine, Mashhad University of Medical Sciences, Mashhad, Iran.*; 2 *Allergy Research Center, Mashhad University of Medical Sciences, Mashhad, Iran. *; 3 *Sinus and Surgical Endoscopic Research Center, Mashhad University of Medical Sciences Mashhad Iran. *

**Keywords:** COVID-19, Hematological indices, Infection, Mucormycosis

## Abstract

**Introduction:**

Rhinosinusitis mucormycosis (RM) is an invasive opportunistic fungal infection, especially among COVID-19 patients. The current study aimed to assess the peripheral blood hematological disorders of COVID-19 patients-associated RM.

**Materials and Methods:**

During ten month, in two COVID-19 centers in Mashhad, Iran, from June 2021 to March 2022, eighty-three patients suspected of COVID-19 with rhinosinusitis or rhino-orbital mucormycosis participated in this study. The hematological indices of these patients were measured by independent sample T‐test or Mann-Whitney test for quantitative data, and the qualitative variables were analyzed using Chi-square or Fisher’s exact test in SPSS version 20 at a significance level of 0.05.

**Results:**

Of the COVID-19 patients, 40 (48.2%) were affected by RM, and leukocytosis due to neutrophilia was observed in 30% of them. Leukocyte counts were normal in 10 (25%) patients, but 1 (2.5%) and 3 (7.5%) had leukopenia and lymphopenia, respectively. Leukocytosis plus lymphopenia was observed in 7 (17.5%) patients. Also, the synchronicity of leukopenia and lymphopenia was seen in 5 (12.5%) patients. Leukopenia, lymphopenia, and neutropenia have occurred concurrently in 2 (5%) patients. The complete blood count (CBC) showed that RBCs, hemoglobin (Hb), hematocrit (HCT), MCH, MCHC, platelet (PLT), and lymphocytes decreased while neutrophils increased.

**Conclusion:**

Among the hematological parameters, leukocytosis due to neutrophilia and reduction in Hb, HCT, and PLT are more dominant factors in COVID-19 patients-associated RM.

## Introduction

Globally, the pandemic of the coronavirus disease 2019 (COVID-19) remains a serious health concern. The way that COVID-19 has manifested itself has been variable,  ranging from moderate  (flu-like symptoms) to severe pneumonia  that involves multiple organs and is potentially fatal (1). Numerous opportunistic bacterial and fungal infections have been linked to it (2-4). Mucormycosis is a rare and invasive fungal infection associated with COVID-19 (2). Mucormycosis is a potentially fatal infection that affects patients with a range of risk factors and manifests clinically in multiple (5). 

The COVID-19 pandemic has recently resulted in a rise in the prevalence of mucormycosis (6). Due to Mucorales’ rapid progression and angioinvasion, they can move from nasal and sinus mucosa into the orbit and brain. According to the rapid progression of rhino-orbital involvement, it is necessary to recognize and treat it to prevent mortality and morbidity (7). 

The class Zygomycetes belongs to the order Mucorales, which are found all over nature, particularly in the soil and decomposing plants. 

The primary factor that appears to promote Mucorales spore germination in COVID-19 patients is an optimal milieu consisting of low oxygen (hypoxia), high glucose (diabetes, steroid-induced hyperglycemia), acidic medium (metabolic acidosis), elevated iron (high ferritin levels), variations in immune blood cell function and count, and reduced phagocytic activity of white blood cells (WBC) (8,9). However, like other invasive filamentous fungal infections, mucormycosis lacks specific clinical or radiological findings (3,10). Consequently, the differential diagnosis of mucormycosis continues to present difficulties, and successful treatment depends on  an early and precise diagnosis (9). Certain results from laboratory tests may be useful to show the patient’s condition and prognosis (11). One of these screening assays is assessing and following-up on hematological disorders via a blood cell count (CBC) test, contributing to better clinical management decisions (8). Most common laboratory findings have shown lymphopenia (11,12). However, comprehensive information about the changes in blood factors inCOVID-19 patients- associated rhinosinusitis mucormycosis (RM) has yet to be available. Therefore, this study was aimed to evaluate the hematologic indices in patients with COVID-19 -associated with RM in northeastern Iran, a country in the Middle East.

## Materials and Methods

This study was approved by the Ethics Committee (ethics code: IR.MUMS. MEDICAL. REC.1400.759). 

The inclusion criteria were patients with COVID-19 with a probable diagnosis of invasive fungal rhinosinusitis, and our exclusion criteria were patients whose rhinosinusitis was not confirmed by laboratory methods. The eighty-three COVID-19 patients with suspected fungal rhinosinusitis were included in this study. 

The patients were admitted to two main COVID-19 Hospital centers (Ghaem and Imam Reza) in Mashhad, Iran (June to March 2022). *All patients* confirmed COVID-19 positivity by real-time *Polymerase Chain Reaction* (*PCR*) and chest computed tomography (CT) scan. Among the suspected patients, forty were confirmed as RM using mycological procedures. RM was confirmed by examining 20% potassium hydroxide (KOH), cultivation on Sabouraud dextrose agar, and morphological identification. Finally, Mucorales species were identified using the molecular method (PCR- Sequencing). Also, histopathological investigation confirmed the mucormycosis. The characteristics of COVID-19-associated patients with RM were recorded, including demographic information and laboratory findings. The hematological indices such as red blood cells (RBC), white blood cells (WBC), and platelets (PLT), as well as hemoglobin (Hb), hematocrit (HCT), mean hemoglobin amount per red blood cell (MCH), and the mean amount of hemoglobin relative to the size of the cell (hemoglobin concentration) per red blood cell (MCHC) were accurately measured and evaluated for any abnormalities. The abnormalities were considered based on the first laboratory examination, compared to the laboratory examination on the day of confirmation of fungal rhinosinusitis. In this study, all analyses were performed using SPSS for Windows, Version 22 (SPSS Inc., Chicago) and Analyse-it for Microsoft Excel (Analyse-it Software, Ltd.). All quantitative variables were expressed as mean ± SD for variables with normal distributions and as median (Percentile 25-75) for variables with a non-normal distribution. The Tannery, and 91 Kirkstall Road, Leeds, United Kingdom). The Kolmogorov‐Smirnov and Shapiro-Wilk tests evaluated the normal distribution of the data before further analysis. Identifier statistics are provided as mean ± SD and median (inter-quartile range, IQR). Intergroup comparisons of clinical and hematological parameters were evaluated by an independent sample t‐test or Mann-Whitney test for quantitative data. Frequency (percentage) was used to describe qualitative variables and compared using Chi-square or Fisher’s exact test. The confidence interval was 95%, and the significance level was P < 0.05.

## Results

Among the 83 COVID-19 patients, 40 patients also showed RM. The patients had an age range of 43–80 years, with a mean age of 62.85 years and an SD of 7.6). Among the 15 patients with diabetes mellitus, 7 (47%) were male and 8 (53%) were female. Additionally, 3 of the 15 patients with diabetes mellitus (20%) died. 

The most common laboratory abnormalities among the patients included leukocytosis (due to neutrophilia) in 12 (30%) cases. Leukocyte counts were normal in 10 (25%) cases, but 1 (2.5%) had leukopenia, and 3 (7.5%) had lymphopenia. Leukocytosis plus lymphopenia was observed in 7 (17.5%). 

**Fig 1:**
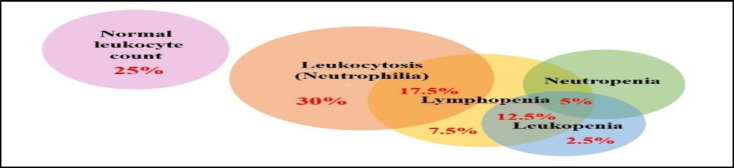
The frequency of white blood cell (WBC) disorders among patients with COVID-19-associated rhinosinusitis mucormycosis

The synchronicity of leukopenia and lymphopenia was seen in 5 (12.5%) of patients, and leukopenia, lymphopenia, and neutropenia occurred in 2 (5%) patients concurrently (Figure1). 

**Fig 2:**
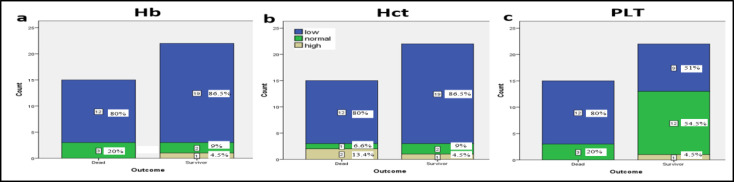
The comparison of hemoglobin (Hb), hematocrit (HCT), and platelet (*PLT*) indices between dead and survivor groups

The CBC results, such as RBCs, PLT, lymphocytes (absolute and percentage), Hb, HCT, MCH, and MCHC were decreased while the neutrophils (absolute and percentage) were increased. They did not show significant differences among the male and female groups (P value: 0.582). The comparison of blood test results in the two groups showed that the average WBC count in males was 9.5 (7.7–14.1) ×**10**^3^**/µL**, the RBC count was 3.7 (3.3–4.3) ×**10**^6^**/µL**, the PLT count was 144 (120–274) ×**10**^3^**/µL** and Hb was 11.5 (9.3–12.4) g/dL. In the female group, the average WBC count was 8.5 (7.5-14.6) ×**10**^3^**/µL,** the RBC count was 3.4 (3.2–3.8) ×**10**^6^**/µL**, the PLT count was 145 (94–219) ×**10**^3^**/µL** and Hb was 10 (9–10.7) g/dL. The differences in the hematological parameters between these two groups are shown in Table 1. The Hb level, HCT%, and PLT count in 80% of dead patients were lower than reference ranges. Also, 86, 87, and 41 percent of patients in the survivor group had low Hb, HCT, and PLT levels, respectively. The differences between the survivor and dead groups were not statistically significant (P value: 0.147) (Figure 2). 

**Table 1:** Hematological parameters in patients with COVID-19-associated rhinosinusitis mucormycosis

Parameters		Total (n = 40)	Male (n = 17)	Female (n = 23)	P value *
Age (years)	Mean ± SD	62.85 ± 7.6	65.7 ± 8.7	60.7 ± 5.9	0.035^**^
WBC (×10^3^/µL)	Median (IQR^#^)	8.6 (7.4–14.9)	9.5 (7.7–14.1)	8.5 (7.5–14.6)	0.891
RBC (×10^6^/µL)	Median (IQR)	3.4 (3.2–4.1)	3.7 (3.3–4.3)	3.4 (3.2–3.8)	0.292
Hb (g/dL)	Median (IQR)	10 (9.1–11.8)	11.5 (9.3–12.4)	10 (9–10.7)	0.151
HCT (%)	Median (IQR)	31.2 (28.3–35.5)	31.8 (28.1–36.6)	30.8 (27.9–32.9)	0.122
MCV (fL)	Mean ± SD	87.7 ± 4.5	89 ± 5.2	86.7 ± 3.8	0.116
MCH (pg)	Mean ± SD	28.5 ± 2.2	29.12 ± 2.6	28.1 ± 1.7	0.193
MCHC (g/dl)	Median (IQR)	32.5 (31.6–33)	32.9 (32–34.5)	32.5 (31–32.9)	0.407
RDW-CV (%)	Median (IQR)	14.7 (13.5–16.3)	14.9 (13.5–15.9)	15.2 (13.7–16.3)	0732
**NEUT** ** (%)**	Median (IQR)	73.3 (66.6–87)	80.6 (72–87.5)	72.7 (69.6–85.4)	0.345
LYMPH (%)	Mean ± SD	16.1 ± 9.9	15.4 ± 10.4	16.7 ± 9.8	0.693
Mix (%)	Median (IQR)	6.1 (4.3–8.5)	6.2 (4–6.5)	5.1 (4.7–9)	0.866
PLT (×10^3^/µL)	Median (IQR)	146.5 (94.5–273)	144 (120–274)	145 (94–219)	0.989
MPV(fL)	Median (IQR)	9.4 (8.3–10.6)	9.1 (8.2–10.6)	9.3 (8.9–10.6)	0.450

Abbreviations: F: Female, M: Male, RBC: Red Blood Cell Count, Hb: Hemoglobin, HCT (%): Hematocrit, MCV: Mean Cell Volume, MCH: Mean Cell Hemoglobin, MCHC: Mean Cell Hemoglobin Concentration, RDW-CV: Red Blood Cell Distribution Width-CV, WBC (%): % of White Blood Cells, NEUT (%): % of Neutrophils, LYMPH (%): % of Lymphocytes, MONO (%): % of Monocytes, EO (%): % of Eosinophils, BASO(%): % of basophils, PLT: Platelet Count, MPV: Mean Platelet Volume, PDW: Platelet distribution width, Mix: contains Monocytes, Basophile and Eosinophil percent. Continuous data were expressed as Mean± SD or Median (Percentile 25-75) for quantitative variables, based on normal or non-normal distribution. *compared to male and female groups, ^**^the differences were significant, ^#^: Interquartile range.

The lowest Hb level, HCT, and PLT count were 6.8 **g/dL**, 21.1 %, 28 ^x^**10**^3^**/μL,** respectively, and belonged to a 56 years old female patient with 6% monocytes, 22% band cells, and also two nucleated RBC (NRBC) that died. Additionally, macrocytic, hypochromic, and rouleaux formations were seen in the peripheral blood smear of a 64-year-old male patient who had diabetes and was discharged with partial recovery.

## Discussion

Invasive fungal diseases are an increasing public health threat and also raise the *risk of* mortality in patients at elevated *risk*. Advances in biomedical and surgical treatments for conditions including cancer, critical illness, transplantation, and HIV have resulted in an expanding population of immunocompromised patients susceptible to fungal infections (2,8, 13). Fungal infections can also complicate other illnesses, as has been clearly illustrated by the impact of mucormycosis on the COVID-19 pandemic. Several studies were reported secondary infections among COVID-19 patients (2-4). According to Jeong et al., diabetes mellitus is a significant risk factor for mucormycosis, as evidenced by a meta-analysis involving 851 patients with mucormycosis (9).

Similarly, diabetes was observed in 37.5% of patients in this current study. Also, Arjun *et al*. describe ten patients affected by rhino-orbital mucormycosis (ROM) from India, and all of them had uncontrolled diabetes (14). In contrast, Veisi et al. reported 2 cases of ROM and found that none had diabetes (15). 

In our study, we observed a higher mortality rate among diabetic patients. Kamat *et al*. analyzed 261 cases of COVID-19-associated *mucormycosis* (*CAM*) affecting the head-and-neck region, with a mortality rate of 25.6% in their systematic review (7). However, despite the growing recognition of the threat of fungal infections, advancements in diagnostics and treatments have not kept pace (7,9). 

The hematology laboratory results are significant because they offer helpful prognostic indicators (11,16). Hence, early diagnosis and aggressive surgical debridement to control the underlying condition can improve the prognosis (1). Patients with phagocyte deficiency or compromised phagocytic function have been shown in multiple studies to be more susceptible to mucormycosis (17). 

To be consumed by phagocytic cells, Mucorales hyphae are too big. Most of this role is played by neutrophils, even though macrophages and monocytes can also harm hyphae. In addition, interactions between several potential oxidative and non-oxidative anti-hyphal mechanisms could characterize the host’s capacity to control fungal infections (11,16,17).

Uncontrolled hyperglycemia could impair phagocytic dysfunction in immune blood cells. Therefore, COVID-19 patients are more likely to be affected by invasive fungal infections (17). Hematological indices such as leukocytes and neutrophil count can be useful indicators for predicting the infection’s severity and predicting the need for additional care (18). 

The incidence of mucormycosis in COVID-19 patients appears to be higher in cases of neutrophilic leukocytosis, severe lymphopenia (more T cells than B cells), inhibitory cytokines, and chemokines when combined with a high and prolonged dose of steroid treatment (6). Jain et al. Pointed out that 65 percent of the 95 post-COVID-19 patients with invasive mucormycosis of the head and neck region had neutrophilic infiltrate with ≤ 50% tissue necrosis (19). Similarly, according to the current study, neutrophilic leukocytosis was more common in COVID-19-associated RM than neutropenia. Observing hematological changes in COVID-19- patients associated with mucormycosis can predict that patients will need further care and assess the risk of serious disease progression. Based on the CBC results, clinicians can make a probable decision according to the patient's clinical signs and underlying diseases (20). Zhang et al. also found that the percentage of neutrophils and lymphocytes has a remarkable diagnostic index. They demonstrated that WBCs, including the percentage and absolute count of neutrophils, were significantly higher in the severe COVID-19 patients, while the percentage and count of lymphocytes and monocyte percentage were significantly lower (21). In line with our current investigation, our data revealed that neutrophilia and lymphopenia were prevalent among the patients. Specifically, we observed an elevation in the absolute neutrophil count. Additionally, the male group exhibited a higher percentage of neutrophils than the female group, although these variances did not reach statistical significance. Conversely, we found a decrease in lymphocyte count. This pattern is in line with the findings of Wang et al., who studied 138 COVID-19 patients. Their results indicated a significantly higher number of peripheral blood neutrophils in the group that died than those that survived (22). Moreover, Hb and HCT are the most notable  and significant indices that have been published in COVID-19 research (23,24). According to a meta-analysis research involving 224 COVID-19 patients in the most critical condition,  Hb levels were considerably lower in severe cases (25). Hb reduction could be a sign of a developing disease. However, age, gender, and underlying disease are also distracting factors in these patients (25). In the study of Henry *et al*., a reduction in Hb levels was observed among 2984 patients (26). In our study, the average Hb level in females was lower than in males. Also, our data revealed that Hb levels are reduced in 65% of males and 91% of females. A meta-analysis study on iron metabolism in COVID-19 patients demonstrated that the median (IQR) RBC count was 9.9 (4.6–14.1)×**10**^6^**/µL**, and Hb was 12.4 (6.5–26.4) g/dL (25). Elevated RBC distribution width (RDW), another parameter of the CBC test, can determine variations of RBC in volume and size. Also, it is an important marker of disease progression and worsening (27). In the study by Aydemir *et al*., a decrease in PLT counts and an increase in MPV values were seen in fungal septic patients (28). In our study, the mean PLT count was lower than the reference range, and the mean platelet volume (MPV) was normal. The present study has some limitations, including a relatively small population. Additionally, we could evaluate a limited number of hospital centers.

## Conclusion

Hematological parameters, particularly leukocytosis driven by neutrophilia, emerged as prominent indicators in patients with COVID-19-associated RM. Additionally, reductions in Hb, HCT, and PLT suggest the potential utility of these parameters as biomarkers in this patient population.
